# Knowledge, Perception, and Associated Factors of Electronic Cigarette Consumption Among the Saudi Population in Jeddah City

**DOI:** 10.7759/cureus.60155

**Published:** 2024-05-12

**Authors:** Abdullah K Qureshey, Abdulrahman F Nawawi, Jehad R Alsaedi, Yazeed A Alzahrani, Hussain A Alkhlaqi, Feras F Filfilan, Abdulrhman M Alghamdi, Mohammed A Aljunaid

**Affiliations:** 1 Medicine, University of Jeddah, Jeddah, SAU; 2 Family and Community Medicine, University of Jeddah, Jeddah, SAU

**Keywords:** jeddah city, saudi population, traditional cigarettes, volatile organic compounds, nicotine, electronic cigarettes

## Abstract

Introduction

Electronic cigarettes (e-cigarettes) are rising in popularity among young adults and teenagers. Previous studies have shown that among high and middle schoolers, the percentage of e-cigarette smokers was noticeably higher than tobacco cigarette smokers. Various research papers focusing on different communities have reported a low-to-moderate level of knowledge and awareness of e-cigarette’s effects on health. E-cigarettes were initially advertised as devices to help people quit smoking, but the use of e-cigarettes in modern days has changed considerably. A big chunk of the population perceived that e-cigarettes have no harmful effects because they are nicotine-free and thus are used as a replacement for regular cigarettes rather than as a way to quit smoking.

Objectives

The study aimed to assess the perception of e-cigarette consumption and associated factors among the Saudi population in Jeddah city.

Methodology

A cross-sectional study was conducted on the Saudi population in Jeddah, Saudi Arabia, during the year 2023. The study assessed the participant’s perceptions of e-cigarette consumption and its risk factors. A pre-existing online questionnaire created by Google Forms was distributed among the population through social media applications to collect data after obtaining their informed consent.

Results

A total of 515 participants were included in this study. Relatives and friends were the most common sources (54%) for information about e-cigarettes. Of the sample, 17.5% thought that e-cigarettes were safer than conventional cigarettes, 13.4% used e-cigarettes on a regular basis, and 65% had not smoked electronically before. Vaping pushed only 17.2% to try traditional tobacco cigarettes, and 25% stopped using traditional tobacco products after starting to smoke e-cigarettes. Gender, age group, and total family salary were the associated factors with the use of e-cigarettes. In addition, an association between the perception of e-smoking and its use was noticed, as well as a significant association between gender and withdrawal symptoms.

Conclusion

A minority of the participants perceived that e-cigarette smoking is safer than conventional methods of smoking. The majority did not practice e-smoking at all. Furthermore, results showed that relatives and friends were the most common sources of information. The findings from the correlation testing underscore several noteworthy associations within the studied population. Notably, gender, age, total family salary, and occupation exhibited statistically significant correlations with e-cigarette usage.

## Introduction

Tobacco use and abuse are considered significant global public health issues [1]. Tobacco smoke can cause severe health issues, including cancer and other serious illnesses because it is loaded with over 7,000 poisonous substances [2]. Many people still smoke or use tobacco products despite the widely recognized health hazards associated with tobacco use [3].

The electronic cigarette (e-cigarette) is a rechargeable battery-powered device that is designed to mimic the sensation of smoking a real cigarette by producing vaporized nicotine that simulates cigarette smoke [4]. It also produces an aerosol that contains fewer toxicants than tobacco smoke. However, prolonged exposure to e-cigarette vapor may lead to nicotine dependence and, consequently, an increased risk of respiratory and cardiovascular health effects [5].

E-cigarettes are thought to have been first introduced into the United States tobacco market in 2007, and despite being advertised as a product to aid in smoking cessation, they captured astonishing numbers of young consumers [6]. Throughout the last 10 years, e-cigarettes have gained much popularity among high school and middle school students as the main source of nicotine consumption [6]. In 2019, a cross-sectional observational study conducted on 19,018 high and middle school students concluded that 27.5% of the high school students surveyed were e‑cigarette users, as opposed to 5.8% reporting conventional tobacco cigarette use. Among middle school students, usage rates were 10.5% and 2.3% for current e-cigarettes and conventional tobacco cigarettes, respectively [7].

In a study from Saudi Arabia, 467 participants who were first-year university students in Riyadh aged between 18 and 24 years showed moderate awareness of the harmful effects of e-cigarettes [8]. Another study conducted in Al-Ahsa, involving 325 participants through a cross-sectional survey of adult male smokers, has shown that the level of knowledge and awareness was high [9]. In a study published in 2022 among the teaching and non-teaching members of King Saud bin Abdulaziz University, the results showed the majority of 337 participants had little knowledge and attitudes toward e-cigarettes [10]. A study among 507 undergraduate students in Thailand showed a lack of knowledge about e-cigarettes’ harmful effects [11].

The use of e-cigarettes is changing, and there is still a lot we do not know about it. The tobacco industry is targeting young adults and promoting their brands as a way to quit regular cigarettes, even though we are not sure about the long-term health effects of e-cigarettes. Most of the research on this topic in Saudi Arabia has focused on medical students, but we are interested in studying the young population and their awareness, associated factors, attitudes, and use of e-cigarettes. Such findings could help regulators make stricter rules. We also want to find out whether adults who only use e-cigarettes are likely to start smoking regular cigarettes. We do not have much evidence about e-cigarette use, its connection to regular smoking, or the factors that influence e-cigarette use in Saudi Arabia. Because it is important to control tobacco use and find safer alternatives, we need to investigate these issues in Saudi Arabia. The World Health Organization considers e-cigarettes toxic and as a way for kids and teens to start smoking, so it is imperative to know how many people are using e-cigarettes and to understand public opinion. The most recent study in Jeddah was done in 2019.

## Materials and methods

Study design

We conducted a cross-sectional survey to assess the perception of e-cigarette consumption and associated factors among the Saudi population in Jeddah City, Saudi Arabia. We used an online questionnaire through Google Forms to gather responses. Participation was voluntary, and the participants’ identities remained anonymous.

Study setting

The study took place in Jeddah, Saudi Arabia, from October to November 2023. It focused on the consumption of e-cigarettes and associated factors among adults living in the city. Ethical approval was obtained at the University of Jeddah (approval no. UJ-REC-166). The consent form was printed on the first page of the questionnaire and stated that by submitting the questionnaire the subject agreed to participate in the study. All information was kept confidential and used only for the purposes of scientific research.

Inclusion and exclusion criteria

The inclusion criteria of this study were adult males and females, above 18 years old, living in Jeddah, who were e-cigarette smokers. Smokers from outside Jeddah city, conventional cigarette smokers, and non-current smokers were excluded. The study excluded those who refused to participate or had any of the following conditions: psychiatric issues or were outside the target age range.

Sample size

According to the online calculator (http://www.raosoft.com/samplesize.html), a sample size of at least 384 is required to accurately represent the population with a 5% margin of error and a 95% confidence level.

Methods for data collection and instrument

A Google Forum e-questionnaire that was distributed among a sample size of 515 participants, appropriate for the latest 2022 Jeddah Census, provided the means for data collection. The questionnaire is a modified version of a pre-existing published questionnaire [12]. The original authors granted permission for the distribution of their questionnaire for the purposes of this research.

Measures

We used the Statistical Package for the Social Sciences (SPSS) to assess the percentage of individuals who were aware of or who used e-cigarettes, the percentage of individuals who stopped traditional smoking habits upon transitioning to vaping, and the percentage of individuals who initiated traditional smoking after beginning the use of e-cigarettes.

Outcome variables

Outcome variables included the awareness and associated factors of e-cigarettes among the general population, the percentage of e-cigarette users within the population, the percentage of individuals who successfully quit smoking as a result of vaping, and the percentage of people who started traditional smoking after starting to vape.

Analyses and entry method

The data will be inputted into a computer using the Microsoft Office Excel software (2016) application designed for Windows. Subsequently, the data will be transferred to the SPSS application for the purpose of conducting statistical analysis (IBM SPSS Statistics for Windows version 20.0, Armonk, NY: IBM Corp.).

## Results

This study was conducted among the Saudi population in Jeddah city; after excluding participants from outside Jeddah city, a total of 515 participants were included in the study. The percentage of males was lower than females (42.7% and 57.3%, respectively). The most common age group was 18-24 years (54.2%). Almost half the participants had a bachelor’s degree (52.2%). The total family salary was less than 75.000 SR per year at 27.6%. Regarding occupation, students constituted (50%) of all respondents, followed by employees at 32.6% (Table 1).

**Table 1:** Sociodemographic characteristics of study participants, Jeddah City (N=515)

Variables	Frequency	Percentage
Gender		
Male	220	42.7
Female	295	57.3
Age groups		
18-24 years	279	54.2
25-34 years	80	15.5
3544 years	56	10.9
45-54 years	64	12.4
55-64 years	31	6.0
65-74 years	4	.8
75 and more	1	.2
Nationality		
Saudi	482	93.6
Non-Saudi	33	6.4
Educational level		
Less than higher school	11	2.1
High school	148	28.7
Diploma	36	7.0
Bachelor’s degree	269	52.2
Master’s degree	34	6.6
Doctorate	17	3.3
Total family salary		
<75.000 SR a year	142	27.6
75.000-150.000 SR a year	115	22.3
150.000-900.000 SR a year	71	13.8
>900.000 SR a year	37	7.2
I don’t know	150	29.1
Current occupation		
Student	261	50.7
Employee	168	32.6
Retired	30	5.8
Not working	35	6.8
Housewife	21	4.1

**Figure 1:**
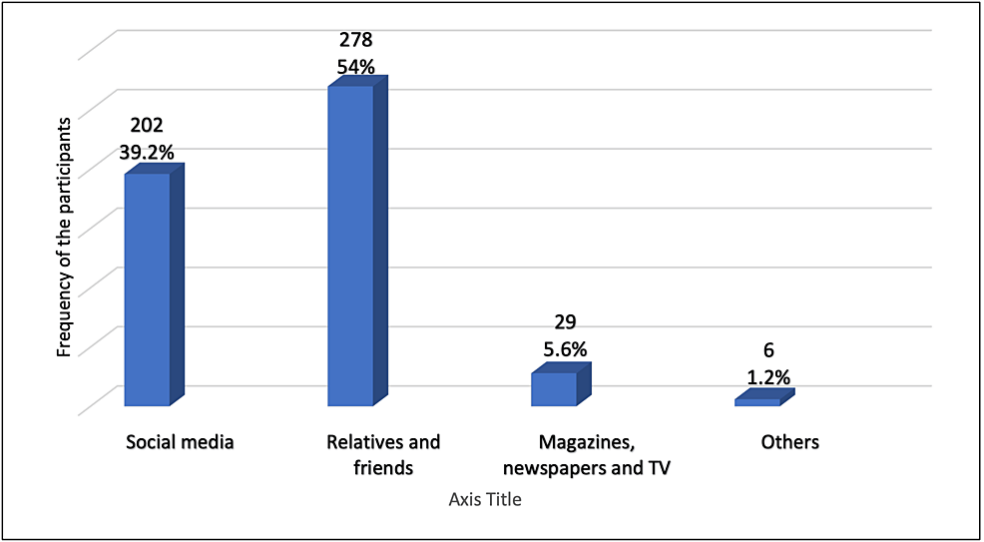
Methods of hearing or learning about e-cigarettes among participants, Jeddah City (N=515) E-cigarette: electronic cigarette

This study included only participants who had heard about electronic smoking and cigarettes previously. The results showed that relatives and friends were the most common source for information about e-cigarettes (54%) followed by social media platforms (39.2%; Figure 1). Many (71.1%) thought the sale and purchase of e-cigarettes is legal and allowed in Saudi Arabia; 44.5% thought that 18 years old is the minimum age requirement for using e-cigarettes, whereas 19.8% agreed that electronic smoking is prohibited for all age groups, and 86% agreed that electronic smoking should be banned in enclosed places or in places where smoking is prohibited, such as workplaces, restaurants, cafes, and cinemas (Table 2). 

**Table 2:** Participants’ beliefs about selling or purchasing e-cigarettes, Jeddah City (N=515) E-cigarette: electronic cigarette

Variable	Answer	Frequency	Percentage
Do you think the sale and purchase of e-cigarettes is legal and allowed in Saudi Arabia?	Yes	366	71.1
	No	48	9.3
	Not sure	101	19.6
Is there a minimum age requirement for using e-cigarettes?	At least 18 years old	229	44.5
	At least 21 years old	76	14.8
	There is no minimum age requirement for electronic smoking	57	11.1
	Electronic smoking is prohibited for all age groups	102	19.8
	Not sure	51	9.9
Do you think electronic smoking should be banned in enclosed places or in places where smoking is prohibited, such as workplaces, restaurants, cafes, and cinemas?	Yes	443	86.0
	No	53	10.3
	Not sure	19	3.7

A small percentage (5.2%) believed that electronic smoking is safer than nicotine patches or nicotine gum; 6.8% thought that e-cigarettes only emit water vapor; 17.5% thought that they are safer than conventional cigarettes and tobacco products, and the source of this information was social media in 28% of cases. Most (70.5%) agreed that e-cigarettes contain dangerous chemicals, but 11.8% believed that the preservatives and flavors used in e-cigarettes are harmless. Only 4.3% believed that exposing children to the use of e-cigarettes is safe, and 56.5% believed that the concentrated nicotine solution in some cartridges or packaging can cause death if swallowed or consumed by a child. Most (79%) thought that electronic smoking in enclosed spaces causes harm to nonsmokers nearby (passive smoking), and only 7.4% considered electronic smoking to be safer for pregnant women and their fetuses. A majority (62.7%) of the participants had family members or friends who practiced electronic smoking (Table 3).

**Table 3:** Participants’ thoughts about the safety and health issues of e-cigarettes, Jeddah City (N=515) E-cigarette: electronic cigarette

Variable	Answer	Frequency	Percentage
Do you believe that electronic smoking is safer than nicotine patches or nicotine gum?	Yes	27	5.2
	No	371	72.0
	Not sure	117	22.7
Do you believe that e-cigarettes and their use are authorized by the Saudi Food and Drug Authority?	Yes	108	21.0
	No	206	40.0
	Not sure	201	39.0
E-cigarettes only emit water vapor.	True	35	6.8
	False	315	61.2
	Not sure	165	32.0
Do you believe that e-cigarettes are safer than conventional cigarettes and tobacco products?	Yes	90	17.5
	No	338	65.6
	Not sure	87	16.9
If you answered Yes or No in the previous question, what is the source of this information?	Friends and relatives	71	13.8
	Social media	144	28.0
	Magazines	9	1.7
	Internet	107	20.8
	Just an assumption	133	25.8
	Others	19	3.7
E-cigarettes contain dangerous chemicals such as nicotine, carbonyls, metals, volatile organic compounds, and fire particles.	True	363	70.5
	False	14	2.7
	Not sure	138	26.8
Do you believe that preservatives and flavors used in e-cigarettes are harmless?	Yes	61	11.8
	No	386	75.0
	Not sure	68	13.2
Do you believe that exposing children to the use of electronic smoke or cigarettes is safe?	Yes	22	4.3
	No	467	90.7
	Not sure	26	5.0
Do you believe that the concentrated nicotine solution in some cartridges or packaging can cause death if swallowed or consumed by a child?	Yes	291	56.5
	No	30	5.8
	Not sure	194	37.7
Does electronic smoking in enclosed spaces cause harm to nonsmokers nearby (passive smoking) due to exposure to chemicals and aerosols emitted from electronic smoking?	Yes	407	79.0
	No	41	8.0
	Not sure	67	13.0
Do you consider electronic smoking and cigarettes to be safer for pregnant women and their fetuses?	Yes	38	7.4
	No	392	76.1
	Not sure	85	16.5
Do any of your family members or friends smoke electronically?	Yes	323	62.7
	No	192	37.3

**Figure 2:**
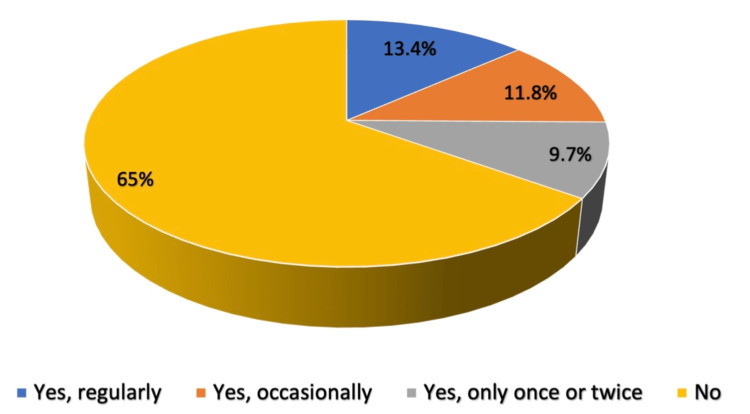
Percentage of participants using e-cigarettes, Jeddah City (N=515) E-cigarette: electronic cigarette

**Figure 3:**
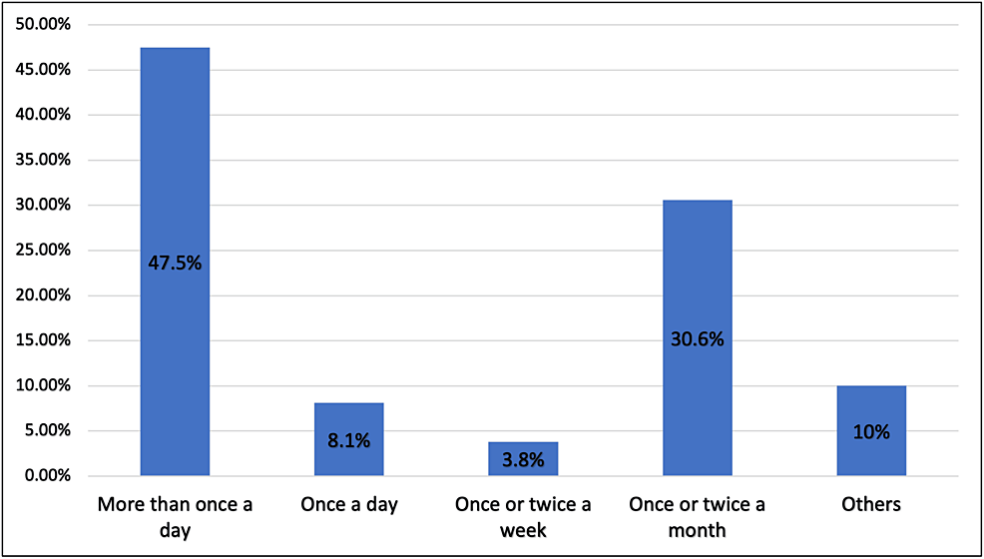
Frequency of e-cigarette use, Jeddah City (N=160) E-cigarette: electronic cigarette

A total of 69 (13.4%) used e-cigarettes or smoked electronically on a regular basis, whereas 61 (11.8%) did so occasionally, and 65% had not smoked electronically before (Figure 2). Almost half (47.5%) used e-cigarettes more than once a day (Figure 3). 

Ease of use was the most common reason that the participants used e-cigarettes (62.8%). The fourth generation of e-cigarettes was the most popular type (48.8%). More than half (55.6%) bought e-cigarettes from tobacco and shisha shops, and 51.1% trusted the nicotine concentration value written on the product’s label. Most of the participants (66.3%) vaped indoors and outdoors (Table 4).

**Table 4:** Usage of e-cigarettes among participants, Jeddah City E-cigarette: electronic cigarette

Variable	Answer	Frequency	Percentage
What attracted you to e-cigarettes?	Ease of use	108	62.8
	Vape volume	14	8.1
	Marketing	6	3.5
	Nice colors	3	1.7
	Fun tricks with the vape	6	3.5
	Small size and modern look	10	5.8
	Others	25	14.5
What is the generation of e-cigarettes that you use?	1st generation	11	6.6
	2nd generation	34	20.5
	3rd generation	20	12.0
	4th generation	81	48.8
	ICOS	3	1.8
	Vape	3	1.8
	Others	14	8.4
Where do you buy e-cigarettes?	E-cigarettes shops	46	27.2
	Tobacco and shisha shops	94	55.6
	Supermarket	9	5.3
	Friends	18	10.7
	Others	2	1.2
Do you trust the nicotine concentration value written on the product’s label?	Yes, I do trust the product’s label	92	51.1
	No, I do not trust the product’s label	47	26.1
	I use nicotine-free cartridges	4	2.2
	Not sure	37	20.6
Where do you vape?	Indoors	22	13.3
	Outdoors	34	20.5
	Indoors and outdoors	110	66.3

**Figure 4:**
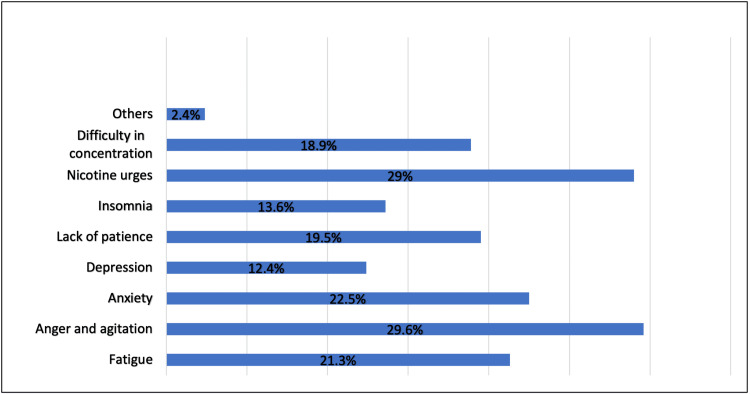
Prevalence of withdrawal symptoms after e-cigarettes use among participants, Jeddah City (N=145) E-cigarette: electronic cigarette

Most participants who smoked (78.9%) experienced withdrawal symptoms after e-cigarette use; the most common withdrawal symptoms were anger and agitation (29.6%), nicotine urges (29%), fatigue (21.3), anxiety (22.5%), lack of patience (19.5%), and difficulty concentrating (18.9%; Figure 4). A small minority (8.3%) had not experienced any withdrawal symptoms because they had not stopped vaping, whereas 36.7% had not experienced any withdrawal symptoms even though they had stopped vaping for a while (Table 5).

**Table 5:** Prevalence of withdrawal symptoms after e-cigarette use E-cigarette: electronic cigarette

Answer	Frequency	Percentage
Yes, I experienced withdrawal symptoms.	145	78.9%
No, I have not experienced any withdrawal symptoms because I have not stopped vaping.	14	3.9%
No, I have not experienced any withdrawal symptoms even though I stopped vaping for a while.	62	17.1%

A few (6.7%) participants went to the emergency department or were admitted to a hospital because of a condition related to vaping; the most common cause of admission was difficulty breathing (30%; Table 6).

**Table 6:** Percentage of participants admitted to hospital because of a condition related to vaping, causes of admission, Jeddah City (N=180)

Variable	Answer	Frequency	Percentage
Have you ever gone to the emergency department or been admitted to a hospital because of a condition related to vaping?	Yes	12	6.7
	No	168	93.3
Cause of admission to hospital	Difficulty in breathing	3	30.0
	Cough	1	10.0
	Dizziness	2	20.0
	Bronchitis	2	20.0
	Loss of sensation	1	10.0
	Others	1	10.0

**Figure 5:**
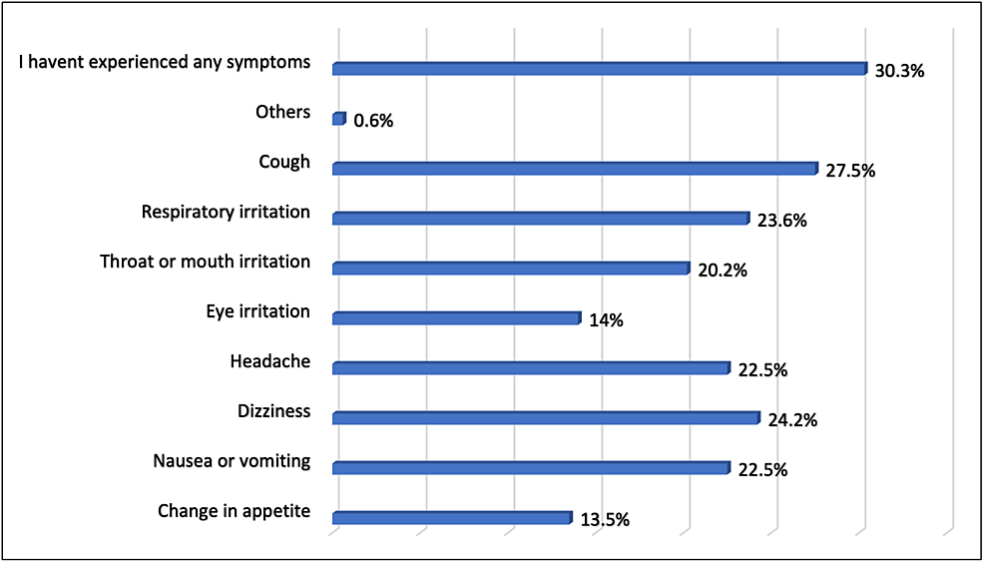
Symptoms experienced after vaping, Jeddah City (N=354)

**Figure 6:**
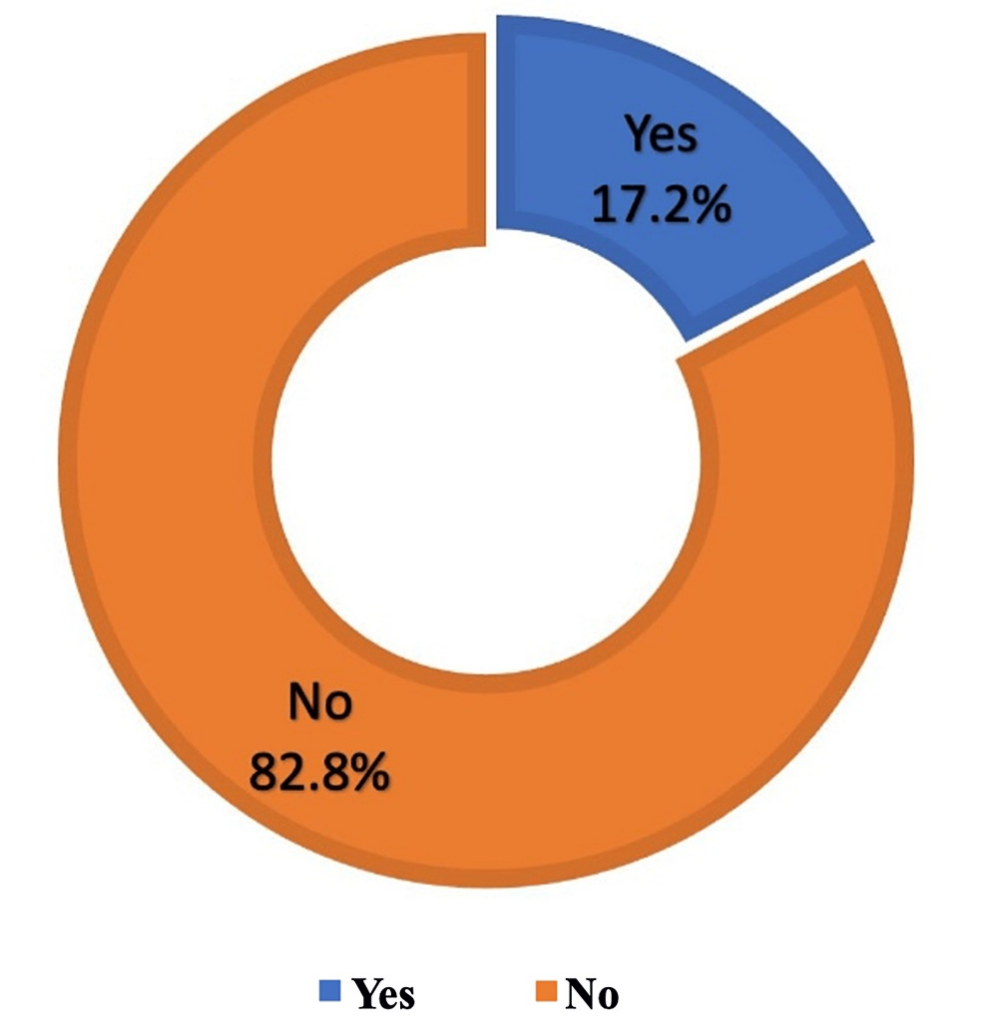
Did vaping push you to try traditional tobacco cigarettes when you had not used them before?

**Figure 7:**
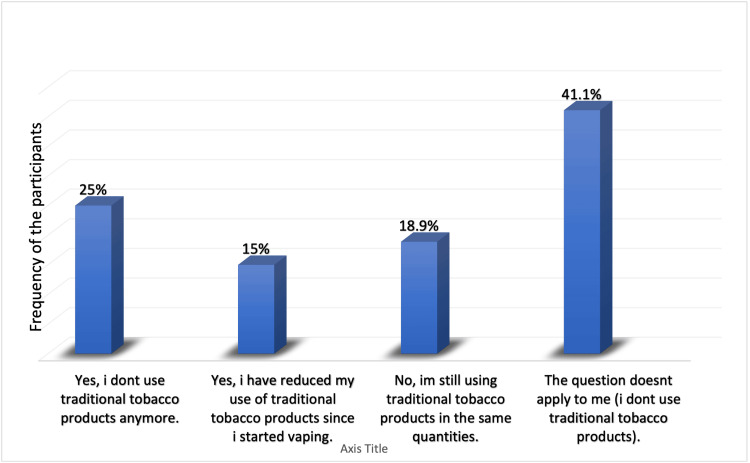
If you vaped or used e-cigarettes to quit traditional tobacco products, did it work? E-cigarette: electronic cigarette

Of the participants who smoked, 84.7% experienced symptoms after vaping; the most common symptoms were cough (27.5%), dizziness (24.2%), and respiratory irritation (23.6%; Figure 5). Results showed that 38.9% had not used traditional tobacco cigarettes (Table 7). Vaping pushed only 31 (17.2%) to try and use traditional tobacco cigarettes (Figure 6), whereas 25% of participants no longer used traditional tobacco products after starting to smoke e-cigarettes, and 15% reduced their use of traditional tobacco products since they started vaping (Figure 7). 

**Table 7:** Percentage of participants who use traditional tobacco cigarettes, Jeddah City E-cigarette: electronic cigarette

Answer	Frequency	Percentage
I have not used traditional tobacco cigarettes.	70	38.9
I am a dual user; I use both traditional cigarettes and e-cigarettes.	48	26.7
I am a previous traditional tobacco smoker; I do not use currently.	62	34.4

Correlation testing revealed a statistically significant association between gender, age, total family salary, occupation of participants, and the use of e-cigarettes (P <.05; Table 8). In addition, Table 9 shows the association between beliefs about e-smoking and its use, where P <.05 is a significant association. Conversely, Table 10 illustrates a significant association between gender and withdrawal symptoms (P-value: <.05). No other variables were significantly associated with the smoking of e-cigarettes (P-value >.05; Tables 11, 12).

**Table 8:** Association between the smoking of e-cigarettes and baseline characteristics of participants, Jeddah City (N=515) E-cigarette: electronic cigarette

Variable	Subgroups	Smokers	Nonsmokers	P-value
Gender	Male	111	109	< .001>
	Female	69	226	
Age group	18-24 years	94	185	.002
	25-34 years	44	36	
	35-44 years	14	42	
	45-54 years	17	47	
	55-64 years	10	21	
	65-74 years	1	3	
	75 and more	0	1	
Nationality	Saudi	163	319	.06
	Non-Saudi	17	16	
Educational level	Less than high school	2	9	.6
	High school	47	101	
	Diploma	14	22	
	Bachelor’s degree	98	171	
	Master’s degree	14	20	
	Doctorate	5	12	
Total family salary	<75 SR a year	56	86	.01
	75.000-150.000 SR a year	39	76	
	150.000-900.000 SR a year	32	39	
	>900.000 SR a year	16	21	
	I don’t know	37	113	
Occupational status	Student	77	184	.004
	Employee	76	92	
	Retired	7	23	
	Not working	15	20	
	Housewife	5	16	

**Table 9:** Association between beliefs about e-smoking and its use

Variable	Subgroups	Smokers	Nonsmokers	P-value
Do you believe that e-cigarettes are safer than conventional cigarettes and tobacco products?	Yes	52	38	< .001>
	No	100	238	
	Not sure	28	59	
Do you think the sale and purchase of e-cigarettes is legal and allowed in Saudi Arabia?	Yes	149	217	< .001>
	No	9	39	
	Not sure	22	79	
Is there a minimum age requirement for using e-cigarettes?	At least 18 years old.	95	134	< .001>
	At least 21 years old.	36	40	
	Not sure	6	45	
	There is no minimum age requirement for e- smoking.	22	35	
Do you think electronic smoking should be banned in enclosed places or in places where smoking is prohibited, such as workplaces?	Yes	127	316	< .001>
	No	44	9	
	Not sure	9	10	
Do you believe that electronic smoking is safer than nicotine patches or nicotine gum?	Yes	15	12	.07
	No	126	245	
	Not sure	39	78	
Do you believe that e-cigarettes and their use are authorized by the Saudi Food and Drug Authority?	Yes	44	64	.2
	No	63	143	
	Not sure	73	128	
E-cigarettes only emit water vapor.	True	19	16	.04
	False	108	207	
	Not sure	53	112	
E-cigarettes contain dangerous chemicals such as nicotine, carbonyls, metals, volatile organic compounds, and fire particles.	True	125	238	.5
	False	7	7	
	Not sure	48	90	
Do you believe that preservatives and flavors used in e-cigarettes are harmless?	Yes	33	28	.004
	No	125	261	
	Not sure	22	46	
Do you believe that exposing children to the use of e-cigarettes is safe?	Yes	12	10	.14
	No	159	308	
	Not sure	9	17	
Do you believe that the concentrated nicotine solution in some cartridges and packaging can cause death if swallowed or consumed by a child?	Yes	94	197	.01
	No	18	12	
	Not sure	68	126	
Does electronic smoking in enclosed spaces cause harm to nonsmokers nearby (passive smoking)?	Yes	124	283	< .001
	No	31	10	
	Not sure	25	42	
Do you consider electronic smoking and cigarettes to be safer for pregnant women and their fetuses?	Yes	23	15	.001
	No	123	269	
	Not sure	34	51	
Do any of your family members or friends use electronic smoking?	Yes	149	174	< .001
	No	31	161	

**Table 10:** Association between sociodemographic characteristics of participants and common withdrawal symptoms

Characteristics	Subgroups	Anger and agitation			Nicotine urge			No withdrawal symptoms		
		No	Yes	P-value	No	Yes	P-value	No	Yes	P-value
Gender	Male	68	41	.001	70	39	.006	80	29	< .001
	Female	53	9		52	10		29	33	
Age group	18-24 years	60	31	.7	58	33	.1	64	27	.3
	25-34 years	29	11		29	11		24	16	
	35-44 years	11	3		12	2		8	6	
	45-54 years	13	3		13	3		7	9	
	55-64 years	7	2		9	0		5	4	
	65-74 years	1	0		1	0		1	0	
Nationality	Saudi	110	44	.6	111	43	.5	97	57	.5
	Non-Saudi	11	6		11	6		12	5	
Educational level	Less than high school	0	2	.005	1	1	.4	2	0	.07
	High school	23	21		28	16		31	13	
	Diploma	10	3		11	2		11	2	
	Bachelor’s degree	73	20		67	26		54	39	
	Master’s degree	10	4		10	4		10	4	
	Doctorate	5	0		5	0		1	4	
Total family salary	<75 SR a year	37	13	.6	35	15	.8	29	21	.7
	75.000-150.000 SR\Y	30	9		30	9		23	16	
	150.000-900.000 SR\Y	20	10		20	10		21	9	
	>900.000 SR\Y	12	4		10	6		11	5	
	I don’t know	22	14		27	9		25	11	
Current occupation	Student	49	26	.6	47	28	.07	52	23	.05
	Employee	52	19		55	16		45	26	
	Retired	6	1		7	0		3	4	
	Not working	10	4		9	5		9	5	
	Housewife	4	0		4	0		0	4	

**Table 11:** Association between sociodemographic characteristics, admission to the emergency department, and common symptoms after vaping

Characteristics	Subgroups	Admission to the emergency department			Respiratory irritation		
		Yes	No	P-value	No	Yes	P-value
Gender	Male	10	101	.1	85	25	.8
	Female	2	67		52	17	
Age group	1824 years	7	87	.5	73	21	.9
	25-34 years	2	42		31	12	
	35-44 years	0	14		12	2	
	45-54 years	1	16		13	4	
	55-64 years	2	8		7	3	
	65-74 years	0	1		1	0	
Nationality	Saudi	10	153	.4	124	38	.9
	Non-Saudi	2	15		13	4	
Educational level		1	1	.007	1	1	.5
	High school	3	44		39	7	
	Diploma	2	12		10	4	
	Bachelor’s degree	3	95		72	26	
	Master’s degree	1	13		11	3	
	Doctorate	2	3		4	1	
Total family salary	<75 SR a year	2	54	.07	43	12	.3
	75.000-150.000 SR\Y	1	38		30	9	
	150.000-900.000 SR\Y	1	31		27	5	
	>900.000 SR\Y	2	14		9	7	
	I don’t know	6	31		28	9	
Current occupation	Student	3	74	.4	58	19	.6
	Employee	7	69		59	16	
	Retired	1	6		5	2	
	Not working	1	14		10	5	
	Housewife	0	5		5	0	

**Table 12:** Association between sociodemographic characteristics and usage of traditional tobacco cigarettes

Characteristics	Subgroups	Use of traditional tobacco cigarettes				Did vaping push you to try traditional smoking?		
		I have not used it	I am a dual user	I am a previous traditional smoker	P-value	Yes	No	P-value
Gender	Male	27	37	47	< .001	18	93	.5
	Female	43	11	15		13	56	
Age group	18-24 years	50	17	27	.001	20	74	.2
	25-34 years	13	12	19		6	38	
	35-44 years	1	9	4		2	12	
	45-54 years	4	8	5		1	16	
	55-64 years	2	2	6		1	9	
	65-74 years	0	0	1		1	0	
Nationality	Saudi	63	44	56	.9	28	135	.9
	Non-Saudi	7	4	6		3	14	
Educational level	2	0	0	.9	0	2	.8
	High school	19	14		14	6		41
	Diploma	5	4		5	2		12
	Bachelor’s degree	38	24		36	20		78
	Master’s degree	4	5		5	2		12
	Doctorate	2	1		2	1		4
Total family salary	<75 SR a year	20	15	21	.2	11	45	.5
	75.000-150.000 SR\Y	15	9	15		4	35	
	150.000-900.000 SR\Y	10	13	9		7	25	
	>900.000 SR\Y	4	5	7		4	12	
	I don’t know	21	6	10		5	32	
Current occupation	Student	45	12	20	< .001	16	61	.4
	Employee	15	33	28		10	66	
	Retired	2	1	4		1	6	
	Not working	7	1	7		2	13	
	Housewife	1	1	3		2	3	

## Discussion

The consequences of e-cigarette use for public health have attracted considerable debate and interest. Evidence-based data about the rate of e-cigarette awareness and use are limited to a few countries [13,14]. However, several previous studies reported a significant increase in awareness and use of e-cigarettes recently [15,16]. Currently, national data on the perception and use of e-cigarettes among the Saudi population are limited, and the research that has been conducted primarily focused on traditional methods of tobacco smoking. There is a need to identify what the public perceives and believes about e-cigarettes as well as who uses e-cigarettes. Accordingly, the current study was conducted to investigate the perception of the Saudi population in Jeddah toward e-cigarettes.

A total of 515 participants were included in the study, out of which more than half were females (57.3%). The most common age group was 18-24 years old (54.2%). This may be explained by the curiosity of youth toward new devices and their higher use of the internet and social media where e-cigarettes are marketed and advertised.

Regarding sources of knowledge about e-cigarettes, results showed that relatives and friends were the most common source of information (54%) followed by social media platforms (39.2%). Similarly, the most common sources reported in previous studies were the internet, friends or personal contacts, and advertisements [17]. Furthermore, a previous study found that older people and lower education groups were more likely to hear about e-cigarettes from television whereas the internet was the main source of knowledge among younger people and higher education groups [18].

Almost half the participants (44.5%) considered that 18 years old is the minimum age requirement for using e-cigarettes, whereas 19.8% agreed that electronic smoking is prohibited for all age groups. In a previous study, it was noticed that younger respondents were more aware of e‑cigarettes than older respondents; the highest percentage of awareness (42.3%) was among the group aged 15-24 years whereas the lowest percentage (6.8%) was among those aged ≥ 55 years [19]. 

Only 5.2% believed that electronic smoking is safer than nicotine patches or nicotine gum, whereas 17.5% assumed that they are safer than conventional cigarettes and tobacco products. This is compatible with the results of a previous study, in which more than one-third of those who are familiar with e-cigarettes (41.6%) thought that e-cigarettes aid in smoking cessation. Meanwhile, 31.9% believed that e-cigarettes are less harmful than traditional cigarettes, and 5.6% reported that e-cigarettes are not harmful at all [18]. In addition, 86% agreed that electronic smoking should be banned in enclosed places or in places where smoking is prohibited, such as workplaces, restaurants, cafes, and cinemas because electronic smoking in enclosed spaces causes harm to nonsmokers nearby (passive smoking).

Regarding the practice of e-smoking, 69 (13.4%) used an e-cigarette or smoked electronically on a regular basis, whereas 61 (11.8%) did so occasionally. Of these, 47.5% used e-cigarettes more than once a day. Ease of use was the most common reason participants were attracted to e-cigarettes (62.8%). The fourth generation of e-cigarettes was the most popular type (48.8%), and 51.1% trusted the nicotine concentration value written on the product’s label. However, 78.9% experienced withdrawal symptoms after e-cigarette use, and the most common withdrawal symptoms were anger and agitation (29.6%), nicotine urges (29%), fatigue (21.3), anxiety (22.5%), lack of patience (19.5%), and difficulty in concentration (18.9%). It is not surprising that most people think e-cigarettes are safer than traditional cigarettes because of the deceptive marketing of the competitive advantages of e-cigarettes over traditional cigarettes.

The findings from the correlation testing underscore several noteworthy associations within the studied population. Notably, gender, age, total family salary, and occupation exhibited statistically significant correlations with e-cigarette usage (P <.05). Furthermore, beliefs regarding e-smoking and its use demonstrated a significant association (P <.05). Interestingly, a noteworthy link between gender and withdrawal symptoms, signifying a statistically significant relationship (P <.05), was observed. These results shed light on the multifaceted interplay among demographic factors, beliefs, and health outcomes in the context of e-cigarette usage.

Based on the findings of this study, we recommend that serial and frequent studies on e-cigarettes should be conducted and funded to generate more evidence and data regarding this topic. Educational campaigns, medical missions, and media recruitment should be held to increase community awareness of this issue, and attempts to correct myths regarding e-cigarettes should be arranged.

Strength of the study

A limited number of Saudi studies have been published in this scope; hence, this study is a valuable base for evidence. Another strength of this study is that it included participants from variable demographic backgrounds and socio-economic levels, which could aid the authorities in dealing with the issue from all aspects. Furthermore, this study paves the way for further studies on this topic.

Limitations of the study

The specific setting of this study may have determined a highly selected group of cases. It may, therefore, be difficult to generalize the findings to the total community in Saudi Arabia. Larger numbers of respondents would have improved the statistical significance of the results.

## Conclusions

A minority of the participants believe that electronic smoking is safer than nicotine patches or nicotine gum and conventional methods of smoking. The majority did not practice electronic smoking at all. Given that individuals’ perceptions affect their behavior, the perception that e-cigarettes used for smoking cessation are less harmful than traditional cigarettes may have multiple impacts, such as a progression to e-cigarette use among non-tobacco users, long-term dual use among current smokers, and relapse of smoking among former smokers; further studies are warranted to provide a significant data to assess the knowledge and perception of using e-cigarettes.
